# Supplementary material for: Assisted reproductive technologies are associated with limited epigenetic variation at birth that largely resolves by adulthood
Source: Nat Commun. 2019 Sep 2;10:3922. doi: 10.1038/s41467-019-11929-9 (PMC6718382; doi:10.1038/s41467-019-11929-9)
Supplement: Supplementary file 1 — Supplementary Information [file 41467_2019_11929_MOESM1_ESM.pdf]

Supplementary Information

Novakovic et al.

Supplementary Figure 1

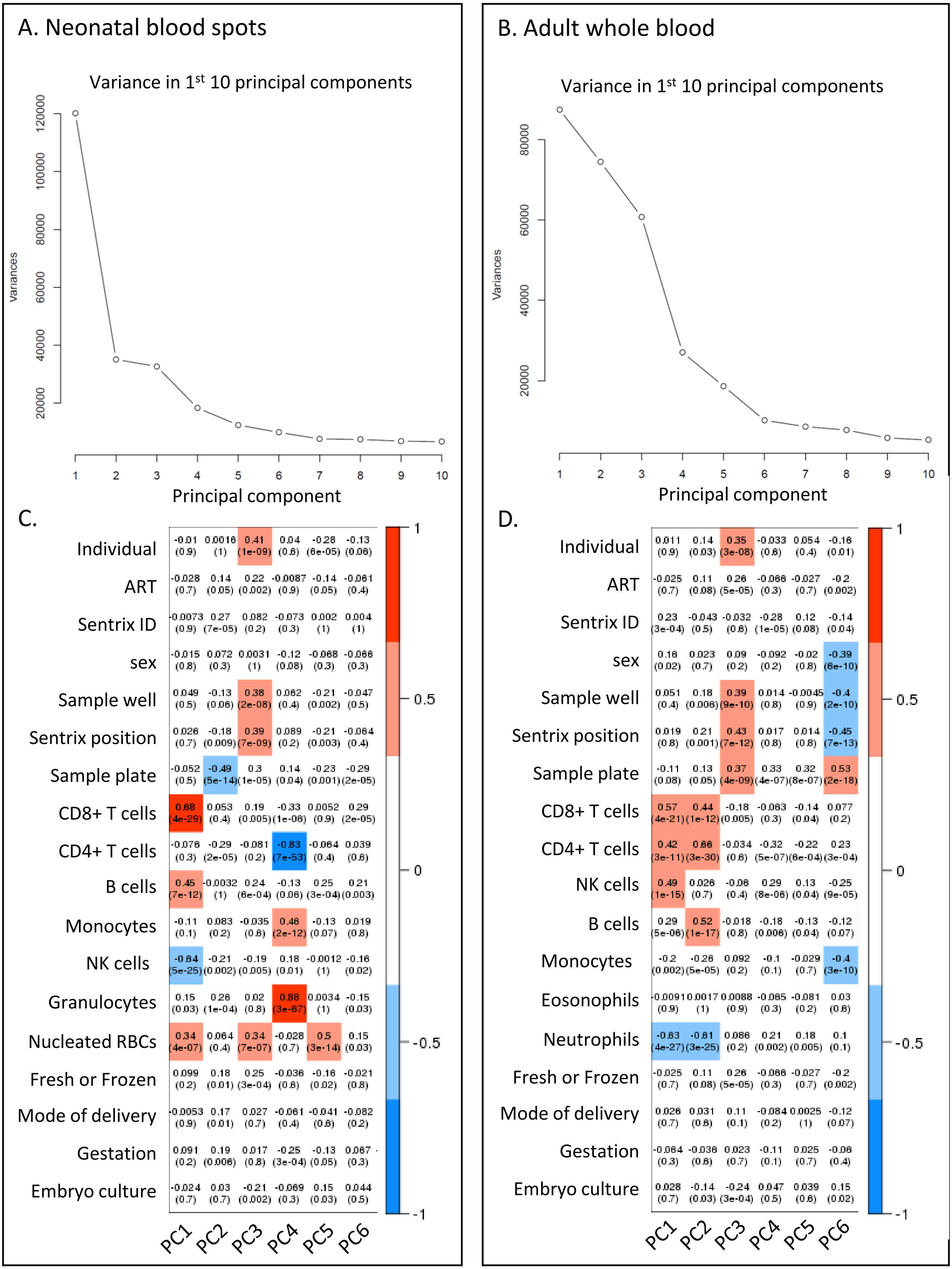

Supplementary Figure 1. Summary of covariates used in the EWAS analysis. PCA loadings in A. neonatal and B. adult blood. C. Heatmap highlighting the contribution of different variables to each principal component in neonatal and D. adult blood. Cell composition was the biggest contributor to variation, while technical variation, such as location of sample in the plate had less of an effect. The top number in each box within the heatmap is the correlation between principal component and data traits and underneath in brackets are the p-values. Covariates with a high correlation and low p-value are highlighted in red for positive correlations and blue for negative correlations.

Supplementary Figure 2

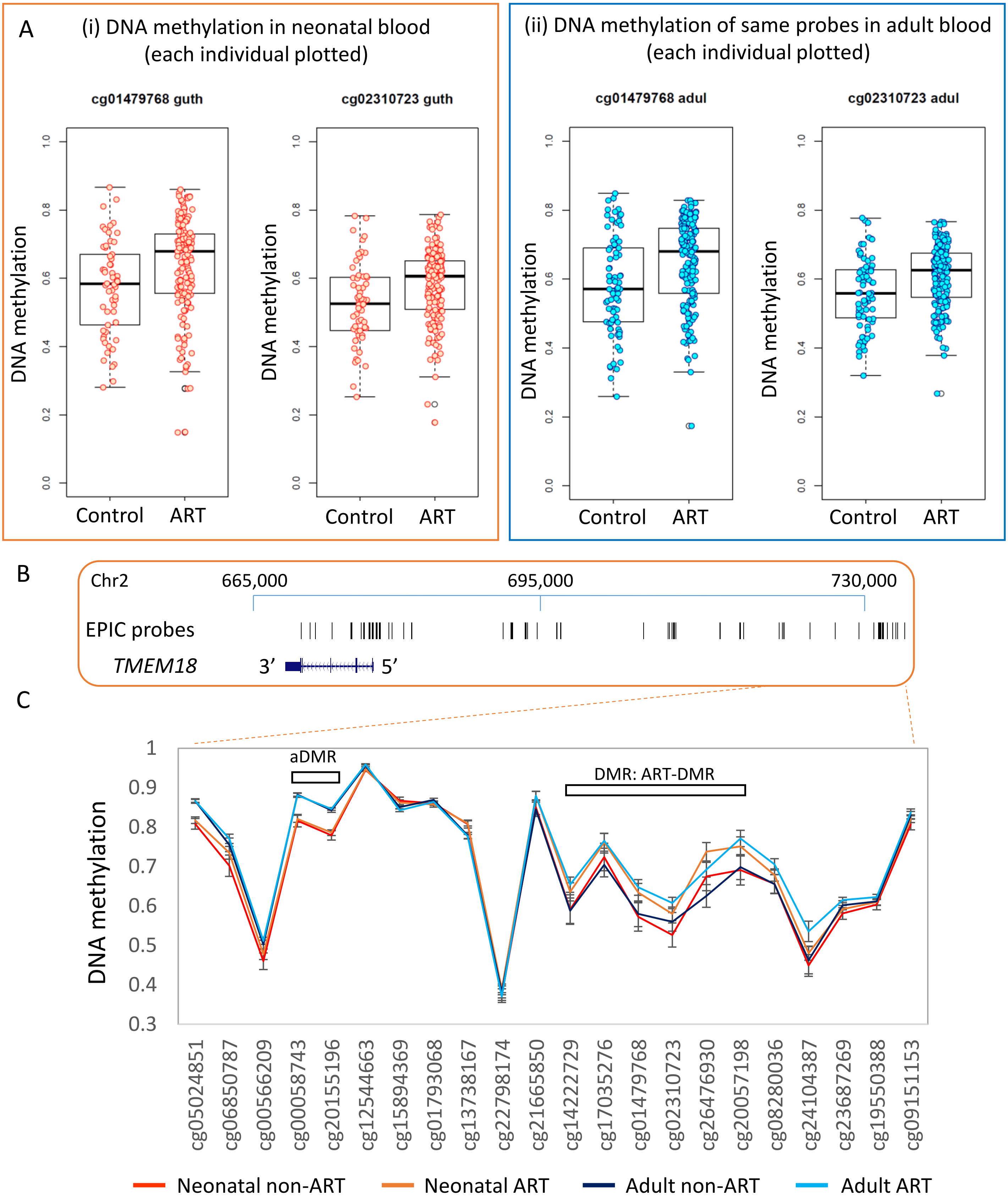

Supplementary Figure 2. Detailed DNA methylation map of the TMEM18 gene. A. (i) Boxplot and dot-plot of DNA methylation for individual neonatal control and ART samples at the two probes within the *TMEM18* gene that showed a difference between groups. (ii) Boxplot and dot-plot of the same two probes in individual adult control and ART samples. The change is no longer significant after correction for multiple testing, but the direction of methylation change persists. Boxplot elements are: center line - median; box limits - upper (Q3) and lower (Q1) quartiles; whiskers – smallest and largest non-outlier; points - outliers. B. Map of the TMEM18 gene in hg19, showing EPIC probe locations. C. Mean DNA methylation level at TMEM18 for neonatal and adult non-ART and ART groups. Error bars are 95% confidence intervals.

Supplementary Figure 3

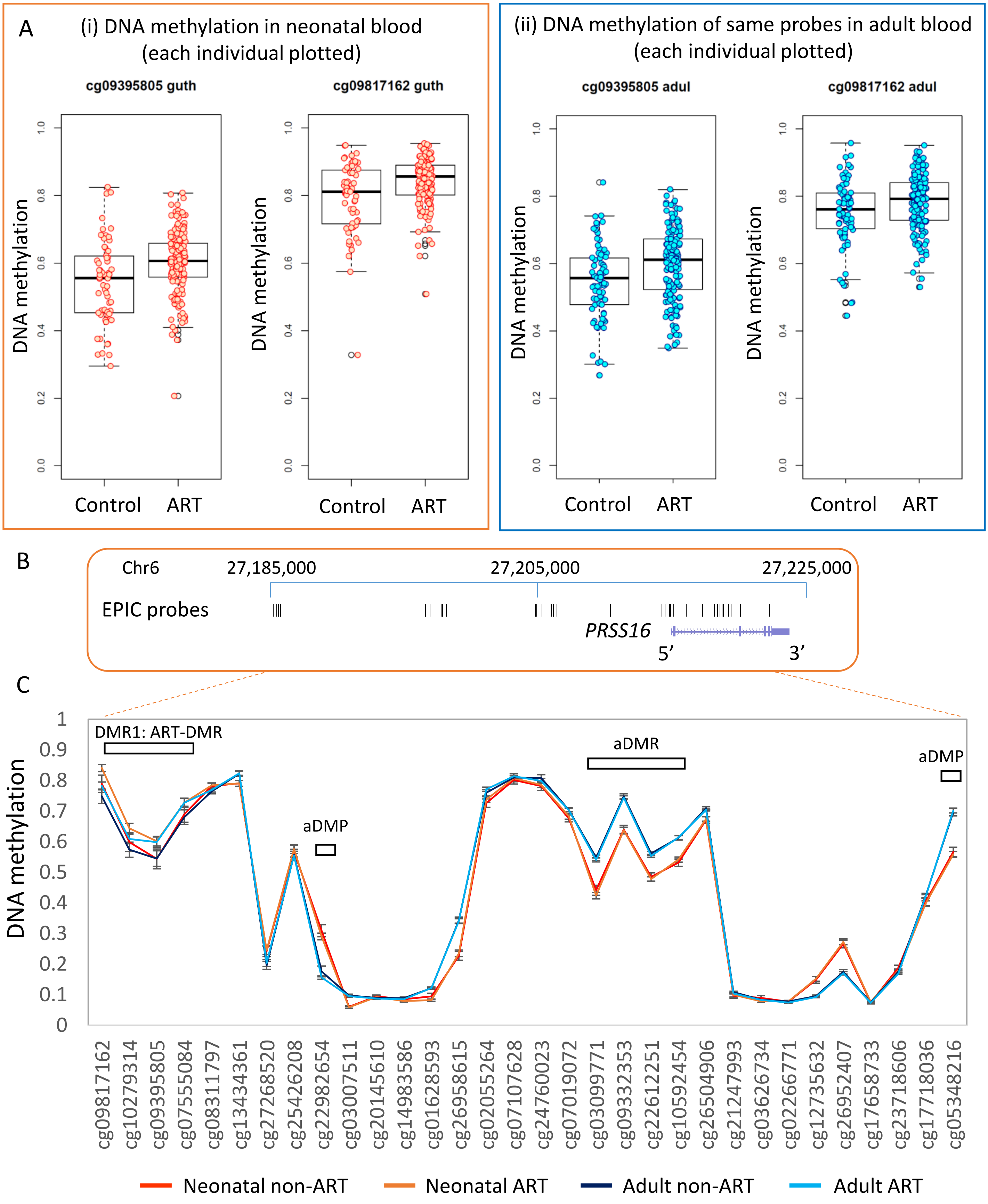

Supplementary Figure 3. Detailed DNA methylation map of the PRSS16 gene. A. (i) Boxplot and dot-plot of DNA methylation for individual neonatal control and ART samples at the top two (out of three significant) probes within the PRSS16 probes that showed a difference between groups. (ii) Boxplot and dot-plot of the same two probes in individual adult control and ART samples. The change is no longer significant after correction for multiple testing, but the direction of methylation change persists. Boxplot elements are: center line - median; box limits - upper (Q3) and lower (Q1) quartiles; whiskers – smallest and largest non-outlier; points - outliers. B. Map of the PRSS16 gene in hg19, showing EPIC probe locations. C. Mean DNA methylation level at PRSS16 for neonatal and adult non-ART and ART groups. Error bars are 95% confidence intervals.

Supplementary Figure 4

A

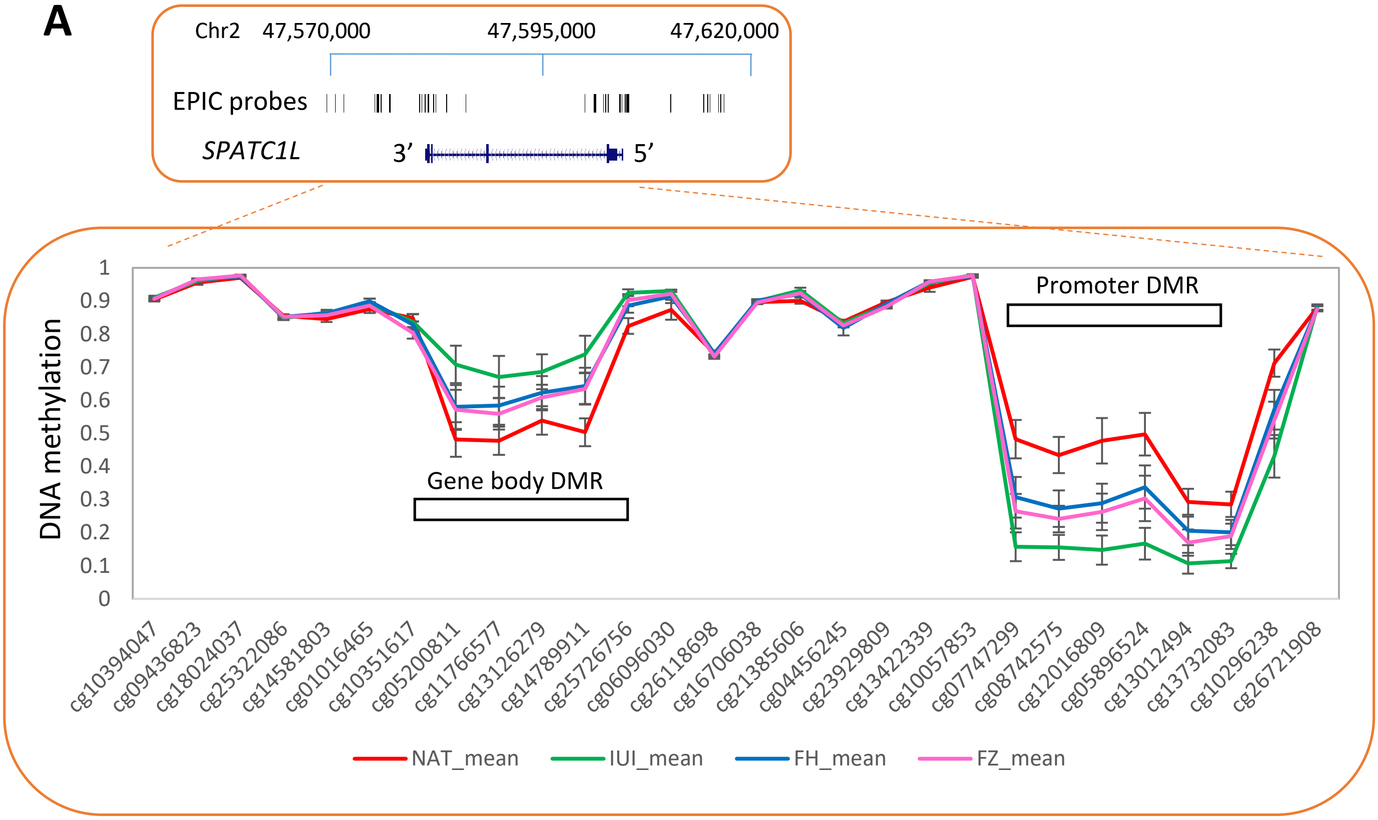

B

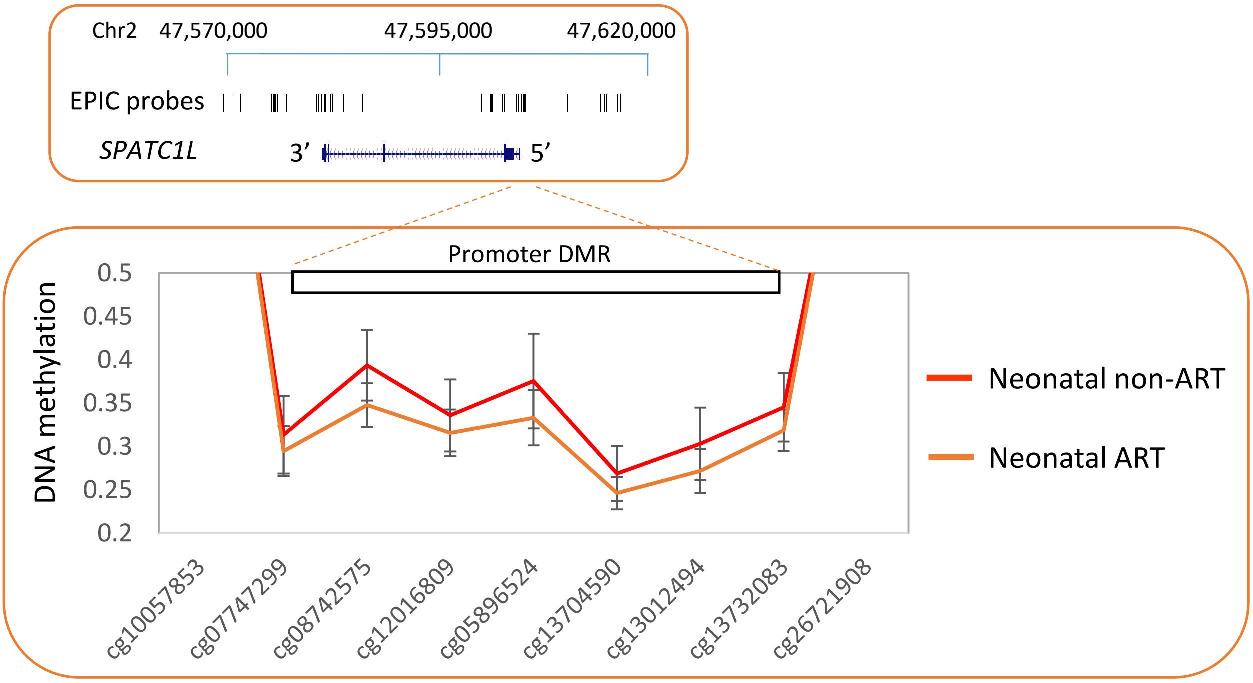

Supplementary Figure 4. Validation of top DMR within SPATC1L gene identified in a previous publication (Estill et al. 2016). A. Map of the SPATC1L gene in hg19, showing EPIC probe locations. B. Mean DNA methylation level at probes within and around SPATC1L in the Estill et al. 450K study. There are two ART-DMRs, one in the promoter and one at the 3' end of the gene. NAT = Natural conception, FH = IVF with fresh embryo, FZ = IVF with frozen embryo, IUI = Intrauterine insemination. B. DNA methylation level in neonatal blood non-ART and ART groups in CHART cohort at the promoter DMR. The change in methylation is in the same direction at previously published, but the effect is lower and does not reach significance after correction for multiple testing.

Supplementary Figure 5

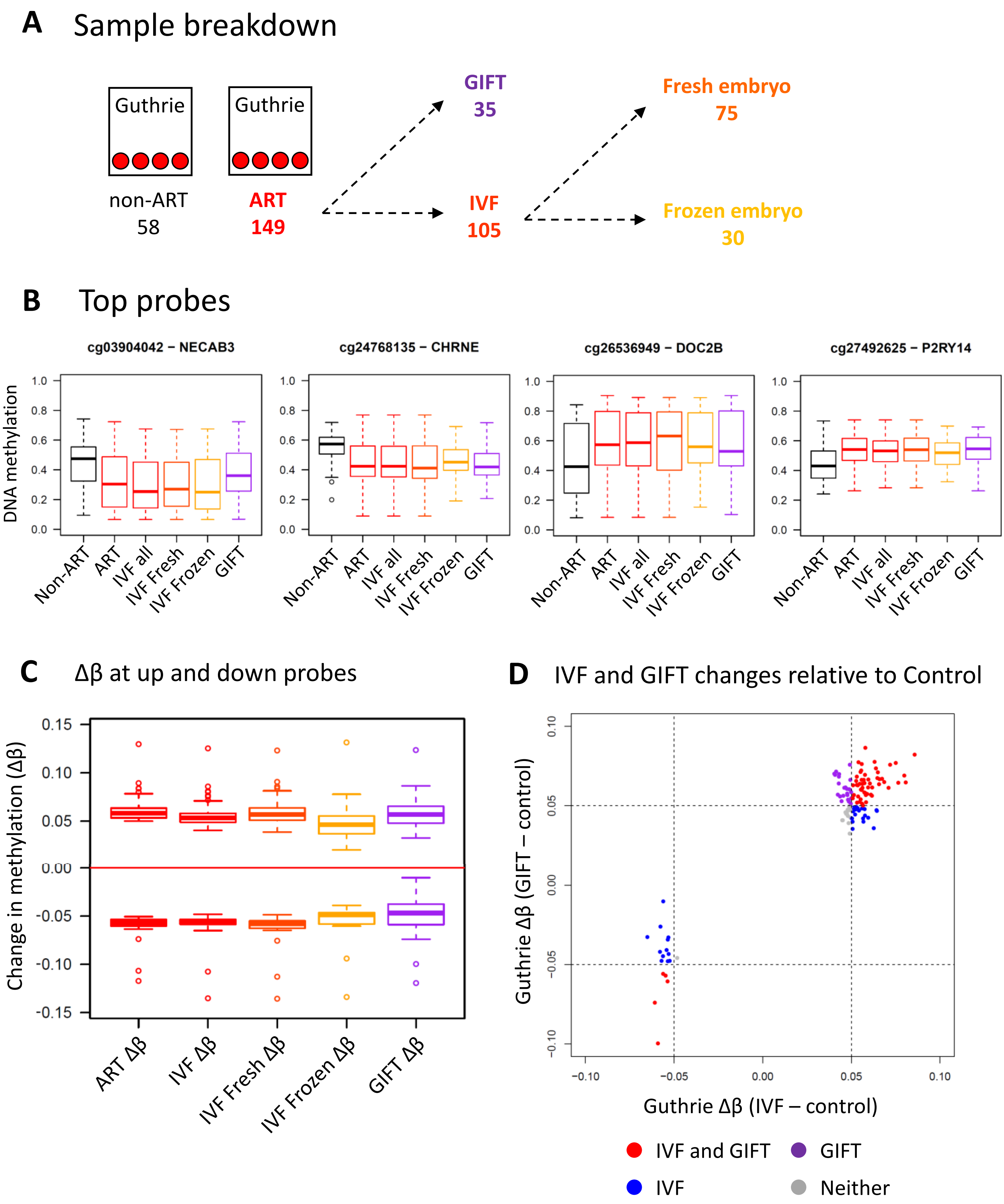

Supplementary Figure 5. DNA methylation levels at neonatal ART-DMRs in different ART subgroups. A. The ART group can be separated into: IVF with fresh embryo, IVF with frozen embryo and GIFT - gamete intrafallopian transfer. B. Distribution of DNA methylation at top probes for each reproductive group that show loss of methylation (associated with CHRNE and NECAB3 genes) or gain of methylation (associated with DOC2B and P2RY14 genes) in neonatal ART group relative to control. A consistent change in methylation is observed in all ART subgroups. C. Boxplot of $\Delta\beta$ values for the 136 significant DMPs identified between neonatal ART and non-ART groups shown for each reproductive group. Boxplot elements are: center line - median; box limits - upper (Q3) and lower (Q1) quartiles; whiskers – smallest and largest non-outlier; points - outliers. D. Correlation between mean change in methylation in the IVF group compared with control (x-axis) and GIFT group compared with control (y-axis). Red: dB >0.05 in both groups, Purple: dB >0.05 only in GIFT group, Blue: dB >0.05 only in IVF group.

Supplementary Figure 6

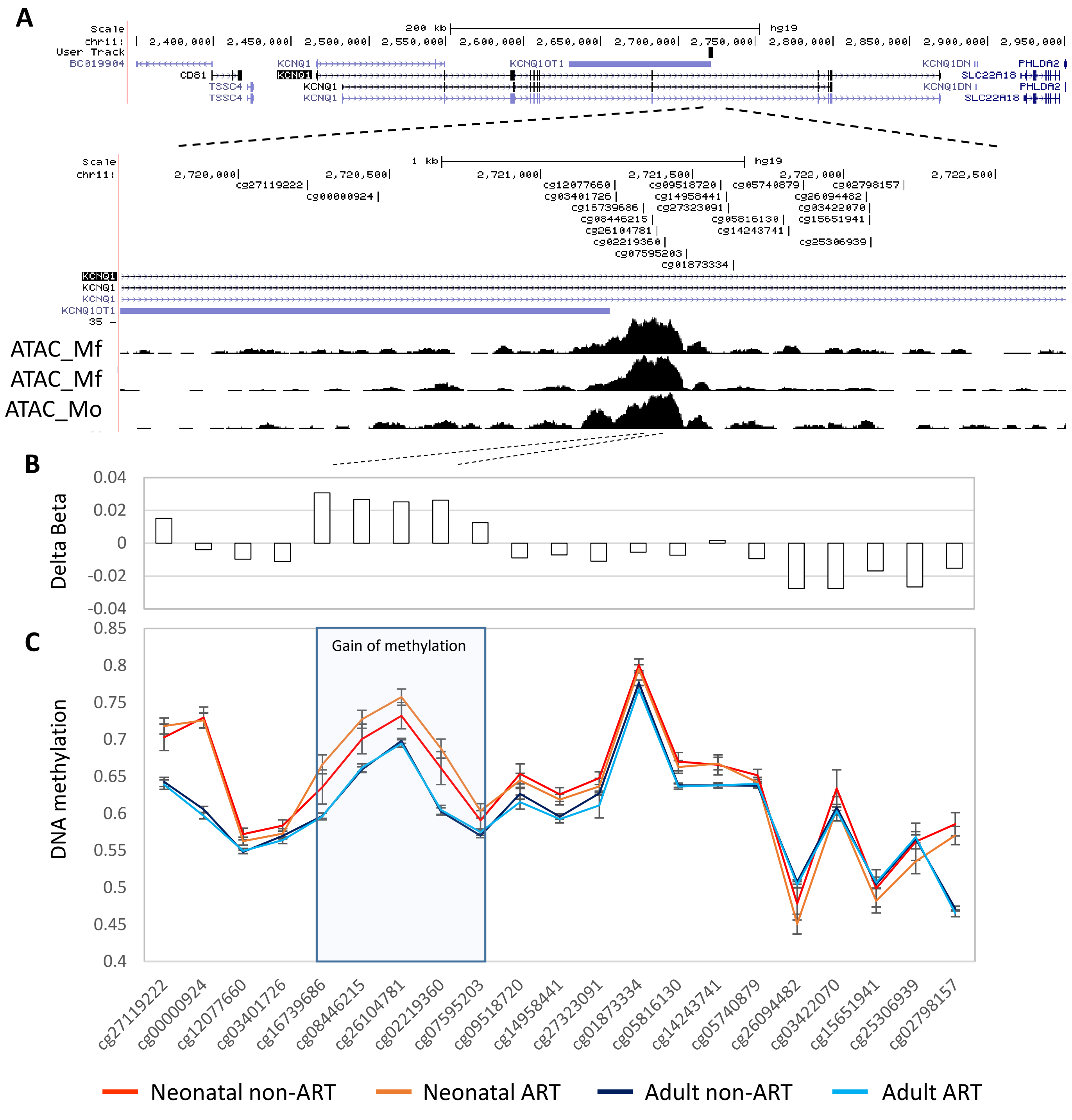

Supplementary Figure 6. Detailed DNA methylation map of the imprinted KCNQ1 gene. A. Map of the KCNQ1 gene in hg19, showing EPIC probe locations. B. Change in DNA methylation at each probe between neonatal ART and control groups. C. Mean DNA methylation level at KCNQ1 for neonatal and adult non-ART and ART groups. Error bars are 95% confidence intervals.
