# Supplementary material for: Assisted reproductive technologies are associated with limited epigenetic variation at birth that largely resolves by adulthood
Source: Nat Commun. 2019 Sep 2;10:3922. doi: 10.1038/s41467-019-11929-9 (PMC6718382; doi:10.1038/s41467-019-11929-9)
Supplement: Supplementary file 3 — Description of Additional Supplementary Files [file 41467_2019_11929_MOESM3_ESM.pdf]

Description of Additional Supplementary Files

File Name: Supplementary Data 1
Description: Significant ART-associated probes in neonates, with annotation, statistics and beta values.

File Name: Supplementary Data 2
Description: Top ART-associated probes in adults, with annotation, statistics and beta values.

File Name: Supplementary Data 3
Description: ART-associated DMRs in neonates, with annotation, statistics and beta values.

File Name: Supplementary Data 4
Description: ART-associated DMRs in adults, with annotation, statistics and beta values.

File Name: Supplementary Data 5
Description: ART-associated probes in neonates with statistics and beta values from the Estill et al. 450K dataset.

File Name: Supplementary Data 6
Description: Common ART-associated DMRs in neonates and adults.

File Name: Supplementary Data 7
Description: Annotation, statistics and beta values of all probes mapped to imprinted regions.
