# Supplementary material for: Assisted reproductive technologies are associated with limited epigenetic variation at birth that largely resolves by adulthood
Source: Nat Commun. 2019 Sep 2;10:3922. doi: 10.1038/s41467-019-11929-9 (PMC6718382; doi:10.1038/s41467-019-11929-9)
Supplement: Supplementary file 11 — Reporting Summary [file 41467_2019_11929_MOESM11_ESM.pdf]

nature research

Corresponding author(s): Richard Saffery

Last updated by author(s): Jul 10, 2019

Reporting Summary

Nature Research wishes to improve the reproducibility of the work that we publish. This form provides structure for consistency and transparency in reporting. For further information on Nature Research policies, see Authors & Referees and the Editorial Policy Checklist.

Statistics

For all statistical analyses, confirm that the following items are present in the figure legend, table legend, main text, or Methods section.

n/a	Confirmed	
☐	☒	The exact sample size (*n*) for each experimental group/condition, given as a discrete number and unit of measurement
☐	☒	A statement on whether measurements were taken from distinct samples or whether the same sample was measured repeatedly
☐	☒	The statistical test(s) used AND whether they are one- or two-sided<br>*Only common tests should be described solely by name; describe more complex techniques in the Methods section.*
☐	☒	A description of all covariates tested
☐	☒	A description of any assumptions or corrections, such as tests of normality and adjustment for multiple comparisons
☐	☒	A full description of the statistical parameters including central tendency (e.g. means) or other basic estimates (e.g. regression coefficient) AND variation (e.g. standard deviation) or associated estimates of uncertainty (e.g. confidence intervals)
☐	☒	For null hypothesis testing, the test statistic (e.g. *F*, *t*, *r*) with confidence intervals, effect sizes, degrees of freedom and *P* value noted<br>*Give P values as exact values whenever suitable.*
☒	☐	For Bayesian analysis, information on the choice of priors and Markov chain Monte Carlo settings
☒	☐	For hierarchical and complex designs, identification of the appropriate level for tests and full reporting of outcomes
☒	☐	Estimates of effect sizes (e.g. Cohen's *d*, Pearson's *r*), indicating how they were calculated

Our web collection on statistics for biologists contains articles on many of the points above.

Software and code

Policy information about availability of computer code

Data collection	The Illumina Infinium MethylationEPIC BeadChip was scanned using the iScan system.<br>Pre-processing of data was performed by a commercial company, using the following packages:<br>MethylAid (http://bioconductor.org/packages/release/bioc/html/MethylAid.html)
Data analysis	The following publicly available packages were used for data analysis:<br>MissMethyl (https://bioconductor.org/packages/release/bioc/html/missMethyl.html)<br>minfi (https://bioconductor.org/packages/release/bioc/html/minfi.html)<br>RStudio (https://cran.r-project.org/; https://www.rstudio.com/)<br>estimateCellCounts (https://www.rdocumentation.org/packages/minfi/versions/1.18.4/topics/estimateCellCounts)<br>limma (http://bioconductor.org/packages/release/bioc/html/limma.html)<br>DMRcate (https://bioconductor.org/packages/release/bioc/html/DMRcate.html)<br>Bedtools (https://bedtools.readthedocs.io/en/latest/)<br>GREAT (http://great.stanford.edu/public/html/)<br>REMP (https://rdrr.io/bioc/REMP/)

For manuscripts utilizing custom algorithms or software that are central to the research but not yet described in published literature, software must be made available to editors/reviewers. We strongly encourage code deposition in a community repository (e.g. GitHub). See the Nature Research guidelines for submitting code & software for further information.

Data

Policy information about availability of data

All manuscripts must include a data availability statement. This statement should provide the following information, where applicable:

- Accession codes, unique identifiers, or web links for publicly available datasets
- A list of figures that have associated raw data
- A description of any restrictions on data availability

The data sets generated and analysed for the current study are deposited in the Gene Expression Omnibus repository with the accession number GSE131433 (https://www.ncbi.nlm.nih.gov/geo/query/acc.cgi?acc=GSE131433). Reviewer password: ixulmkqazduvtgn

Field-specific reporting

Please select the one below that is the best fit for your research. If you are not sure, read the appropriate sections before making your selection.

☒ Life sciences ☐ Behavioural & social sciences ☐ Ecological, evolutionary & environmental sciences

For a reference copy of the document with all sections, see nature.com/documents/nr-reporting-summary-flat.pdf

Life sciences study design

All studies must disclose on these points even when the disclosure is negative.

Sample size	DNA methylation status was generated for 149 neonatal (84 female, 65 male) and 158 adult (87 female 71 male) ART-conceived individuals and for 58 neonatal (37 female, 21 male) and 75 adult (51 female, 24 male) non-ART conceived individuals. This represents all ART individuals and controls recruited by the CHART cohort. This allows for detection of differentially methylated probes after correction for multiple testing.
Data exclusions	Samples were checked for quality and those with a mean detection p-value of >0.01 were removed (5 neonatal and 4 adult samples), leaving 207 neonatal blood (n=149 ART, n=58 non-ART) and 233 whole adult blood (n=158 ART, n=75 non-ART) samples for analysis.
Replication	Significant probes were validated in an unrelated neonatal cohort of 94 ART individuals and 43 non-ART individuals. The second study was performed with the HumanMethylation450 platform, which covers 36% of the significant EPIC array probes. Importantly, 2 out of 3 differentially methylated regions identified in our study were replicated in the second cohort.
Randomization	Because the study represents the complete CHART cohort, there was no randomization of individuals, other than the initial matching of cases and controls during recruitment. In order to avoid technical variation, the samples were randomly distributed across Illumina Infinium MethylationEPIC array chips.
Blinding	DNA extractions were performed by research assistants that were blinded to the groups. Analysis of data was not blinded, because it was necessary to split the samples into two groups - ART and non-ART.

Reporting for specific materials, systems and methods

We require information from authors about some types of materials, experimental systems and methods used in many studies. Here, indicate whether each material, system or method listed is relevant to your study. If you are not sure if a list item applies to your research, read the appropriate section before selecting a response.

Materials & experimental systems

n/a	Involved in the study	
☒	☐	Antibodies
☒	☐	Eukaryotic cell lines
☒	☐	Palaeontology
☒	☐	Animals and other organisms
☐	☒	Human research participants
☒	☐	Clinical data

Methods

n/a	Involved in the study	
☒	☐	ChIP-seq
☒	☐	Flow cytometry
☒	☐	MRI-based neuroimaging

Human research participants

Policy information about studies involving human research participants

Population characteristics	Participants were adults aged 22-35 years conceived with and without use of ART in the State of Victoria, Australia. A total of 193 ART- and 86 non-ART- conceived adults participated (ART: 80 Male, 113 Female; Non-ART: 28 male, 58 female). The mean age (SD) was comparable in both groups (27.5 (2.8); 27.6 (2.6)). Participants consented to providing a venous blood sample and researcher access to previously collected neonatal blood spots for DNA isolation and epigenetic analysis. DNA methylation status

	was generated for 149 neonatal (84 female, 65 male) and 158 adult (87 female, 71 male) ART-conceived individuals and for 58 neonatal (37 female, 21 male) and 75 adult (51 female, 24 male) non-ART conceived individuals
Recruitment	Originally, the mothers of the young adults (ART-conceived and non-ART-conceived) were recruited with the use of random digit dialing in the general population, identifying and screening households for those with a consenting mother of a young adult aged 18–28 years. Recruitment continued until there were approximately equal numbers of ART and non-ART young adults. Mothers and the young adults were both interviewed by telephone and provided health information. The young adults from this original interview study were contacted to consent to the current clinical review/epigenetic study. Some individuals in both the ART and non-ART groups, with adverse health conditions, may not have participated in the current study and this is mentioned as a limitation to the study, i.e. the possibility that there has been a healthy participant bias. This may have impacted on the magnitude of the methylation differences, with greater effects seen in those less healthy, but, overall non-differential bias would not alter the results demonstrating differences between ART and non-ART participants.
Ethics oversight	The study was approved by The Royal Children's Hospital Human Research Ethics Committee (RCH HREC Project 33163)

Note that full information on the approval of the study protocol must also be provided in the manuscript.
